# FGF/FGFR system in the central nervous system demyelinating disease: Recent progress and implications for multiple sclerosis

**DOI:** 10.1111/cns.14176

**Published:** 2023-03-16

**Authors:** Qingxiang Zhang, Zhiguo Chen, Kaili Zhang, Jie Zhu, Tao Jin

**Affiliations:** ^1^ Neuroscience Center, Department of Neurology The First Hospital of Jilin University Changchun China; ^2^ Cell Therapy Center, Beijing Institute of Geriatrics, National Clinical Research Center for Geriatric Diseases Xuanwu Hospital, Capital Medical University Beijing China; ^3^ Key Laboratory of Neurodegenerative Diseases Ministry of Education Beijing China; ^4^ Stomatology College of Inner Mongolia Medical University Hohhot China; ^5^ Department of Neurobiology, Care Sciences & Society Karolinska Institute, Karolinska University Hospital Solna Stockholm Sweden

**Keywords:** fibroblast growth factor receptors, fibroblast growth factors, inflammation, multiple sclerosis, remyelination

## Abstract

**Background:**

With millions of victims worldwide, multiple sclerosis is the second most common cause of disability among young adults. Although formidable advancements have been made in understanding the disease, the neurodegeneration associated with multiple sclerosis is only partially counteracted by current treatments, and effective therapy for progressive multiple sclerosis remains an unmet need. Therefore, new approaches are required to delay demyelination and the resulting disability and to restore neural function by promoting remyelination and neuronal repair.

**Aims:**

The article reviews the latest literature in this field.

**Materials and methods:**

The fibroblast growth factor (FGF) signaling pathway is a promising target in progressive multiple sclerosis.

**Discussion:**

FGF signal transduction contributes to establishing the oligodendrocyte lineage, neural stem cell proliferation and differentiation, and myelination of the central nervous system. Furthermore, FGF signaling is implicated in the control of neuroinflammation. In recent years, interventions targeting FGF, and its receptor (FGFR) have been shown to ameliorate autoimmune encephalomyelitis symptoms in multiple sclerosis animal models moderately.

**Conclusion:**

Here, we summarize the recent findings and investigate the role of FGF/FGFR signaling in the onset and progression, discuss the potential therapeutic advances, and offer fresh insights into managing multiple sclerosis.

## Graphical abstract

Fibroblast growth factors are involved in the MS development and crucial processes.

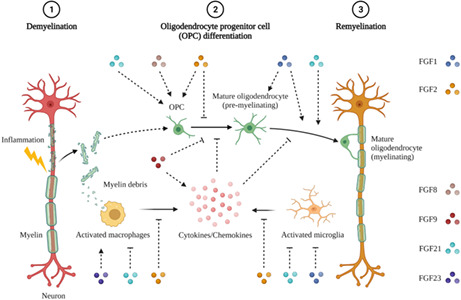

## 1 INTRODUCTION

Multiple sclerosis (MS) is a chronic demyelinating condition of the central nervous system (CNS) caused by lymphocytic infiltration, damaging axons, and their associated myelin sheath.^1^, ^2^ It is the leading cause of non‐traumatic neurological disorders in young adults and causes significant disability, and high direct and indirect costs worldwide. Dysregulated immunity, genetic predisposition, and environmental variables interact in a complicated way to cause MS.^1^ Vitamin D insufficiency, related to reduced exposure to ultraviolet type B light, is the main environmental factor. The risk of MS is higher for adolescent patients living in northern or southern latitudes than those in equatorial regions.^3^ Approximately 85% of MS cases begin with episodic neurological impairment involving the brainstem, optic nerve, and spinal cord, which resolves spontaneously, is termed relapsing–remitting MS (RRMS), and mainly affects individuals in early adulthood.^1^ Approximately 20% of patients develop symptoms after age 40, and disability progression in the male population with late onset is faster than in young RRMS patients.^4^ In 50% to 60% of RRMS cases, the condition develops into secondary progressive MS (SPMS), characterized by an infrequent or completely terminated relapse after 10–15 years with a slow progression of irreversible disabilities associated with neurodegeneration.^5^, ^6^ Unidirectional progressive disability from the outset is observed in 15% of MS cases and is referred to as primary progressive MS (PPMS). For this patient cohort, the rapid onset of early neurodegeneration is the best predictor of long‐term progression rates.^6^ In addition to experimental autoimmune encephalomyelitis (EAE), the most frequently employed model,^7^ several demyelinating models have also been used in MS research. Both clinical and pathological characteristics of human MS are shared by the murine hepatitis virus (MHV) strain A59 that causes demyelination in animal models,^8^ and lysophosphatidylcholine has been used to induce inflammatory demyelination, in which the myelin structures, as well as the blood–brain barrier (BBB), are disrupted, but neuronal loss is absent.^9^ Moreover, the chronic cuprizone demyelination model leads to consistent demyelination followed by spontaneous remyelination within a short period.^10^ Thus, these models are valuable supplements to EAE research and are appropriate for studying the mechanisms of demyelination and therapeutic interventions for MS.

The fibroblast growth factor (FGF) family contains 23 members, including 18 protein ligands (the murine FGF15 and the human FGF19 genes are orthologous) and four fibroblast homologous factors (FHFs). FGF ligands exercise their functions by binding with their high‐affinity receptor family (FGFR1‐4) and are involved in fundamental physiological processes in adults, including wound repair, angiogenesis, and metabolism.^11^, ^12^ The roles of FGF/FGFR signaling on essential cellular function control indicates the relevance of this axis in the pathogenesis of MS.^13^, ^14^ Over the past few years, interventions targeting FGF/FGFR have moderately ameliorated symptoms in animal models, and the conditional deletion of FGFR1 and FGFR2 has shown remarkable therapeutic promise.^15^, ^16^ Furthermore, several FGF family members are strongly associated with the pathogenesis and course of MS.^17^, ^18^, ^19^ Here, we summarize the latest relevant findings, discuss the function of FGF/FGFR signaling in MS pathogenesis, and describe potential therapeutic advances, providing fresh perspectives on MS therapy.

## 2 UNDERSTANDING THE PATHOGENESIS OF MS

### 2.1 Inflammation

Multiple sclerosis is a chronic inflammatory demyelinating and degenerative condition of the CNS. Inflammation of the spinal cord and brain is invariably present in all phases of MS and declines with disease progression.^20^ A dominant aspect of the early pathology in RRMS patients is active inflammatory demyelinating lesions, which arise through inflammatory infiltrates associated with disrupted BBB.^21^ At this stage, lesions of relapsing MS have more plentiful macrophages than any type of progressive MS (PMS) to phagocytose the myelin degradation products.^22^ In contrast, chronic lesions predominate in progressive disease. Lymphocyte infiltration is initially blocked in the leptomeninges and blood vessels behind an intact BBB.^23^ Here, T cells attract immune cells into the CNS by interacting with major histocompatibility complex class II^+^ microglia^24^ and produce adhesion molecules, chemokines, and a variety of proinflammatory cytokines.^20^ Another type of inflammation is densely populated by B cells of the brain's connective tissue spaces adjacent to an intact BBB, where they may form aggregates or tertiary lymph follicles.^21^, ^25^ Interestingly, the inflammatory state of the CNS might provide a favorable environment for lymphocyte proliferation and expansion.^20^ Furthermore, mononuclear phagocytes (MPs), namely resident microglia and the macrophages differentiated from infiltrating monocytes, also act as a significant part in the pathologic mechanisms of PMS. According to experimental and clinical studies, MPs present in demyelinating lesions secrete chemokines that induce lymphocytes to infiltrate the CNS and thus provide an inflammatory environment. They also generate reactive oxygen and nitrogen species, which leads to oligodendrocyte and neuronal cell death.^1^, ^26^, ^27^ In addition, CNS glial cells may initiate an immunological response in MS (particularly in PMS, as it is intimately linked to the chronic activation of the innate immune system).^28^ Some studies have pointed out that, at least in certain situations, MS may originate from a primary injury within the CNS, possibly associated with oligodendrocytes, followed by glial activation and ultimately by immune‐mediated inflammatory activation as a secondary response.^25^

### 2.2 Demyelination and neurodegeneration

Demyelination leads to a reduction in axonal integrity and, over time, to neuronal dysfunction. Neurodegeneration is a characteristic of MS and a major contributor to clinical impairment and decreased quality of life. Demyelinated axons become frangible and suffer damage from activated immunological and glial cells releasing cytokines, oxidative products, and free radicals. Even in the predominantly inflammatory demyelinating stage of the disease, transected axons are abundant, demonstrating that axonal loss occurs at disease onset and continues with time. In the initial phases of RRMS, the axonal loss has no immediate substantial clinical impact. As lesions accumulate with time, however, the clinical aspects of MS become driven by axonal loss. Thus, it is believed that the brain's ability to adjust for further axonal loss exhausts before RRMS and SPMS shift. At this stage, MS lesions include remyelination, inflammation resolution without repair, or a “smoldering” state of coexistence of inflammation and myelin degeneration.^29^ In PMS, depletion of the myelination capacity by both oligodendrocyte precursor cells (OPCs) and residual oligodendrocytes is critical. Recent studies of MS using human single‐nucleus RNAseq demonstrated that oligodendrocytes respond rapidly to oxidative stress, with the downregulation of homeostasis and myelin synthesis genes.^30^ Moreover, when the dynamics of oligodendrocyte generation in MS brain tissue were assessed by ^14^C methods, it was found that the demyelination is partly caused by the depletion of the myelination ability of the surviving oligodendrocytes rather than by an impairment in OPC differentiation.^31^ These results may impact the establishment of disease models and the development of myelin regeneration strategies for PMS. RRMS has become a pharmacologically treatable condition. However, PMS continues to face treatment challenges because of the persistent accumulation of neurological impairments and disabilities.

### 2.3 Remyelination failure

Demyelination can occur parallel to regeneration processes, which restore some of the destroyed myelin‐generating cells and rebuild the myelin sheath around axons. This is accomplished by the activation, migration, and polarization of resident OPCs and neural stem cells (NSCs) into myelin cells, initiating an oligodendrocyte‐driven repair process known as remyelination. The identity of cells that cause remyelination in the CNS of MS patients has been a subject of debate. OPCs and mature oligodendrocytes that have survived are two potential candidates. This discussion is crucial because therapeutic approaches to enhance remyelination may differ depending on the specific cellular pathways involved. Lineage tracing experiments revealed that newly generated oligodendrocytes derived from OPCs form new myelin sheaths in demyelinated regions.^32^ However, it has also been found that surviving oligodendrocytes can expand and remyelinate axons in MS.^31^ Moreover, myelin sheaths derived from OPCs are thinner and less functional than those generated by the surviving oligodendrocytes. Myelin cells in the adult CNS can also differentiate from NSCs in the subventricular region. The microenvironment of the demyelinating lesions substantially impacts OPC and NSC homeostasis, in addition to uncontrollable factors such as gender and age and may also be the target of future remyelination treatment strategies. Other glial cells, like microglia, are momentous to remyelination and aid in removing myelin debris and releasing neurotrophic factors that support OPC functions.^33^, ^34^ Among them, CX3CR1, a fractalkine receptor that is abundantly expressed on microglia, has been shown to affect the ability of these cells to phagocytose. Reduced microglial phagocytosis in cuprizone‐treated CX3CR1‐deficient animals causes a continuous accumulation of myelin debris, inhibiting remyelination due to insufficient OPC recruitment.^35^ Additionally, the disequilibrium of pro‐regeneration and inhibitory elements limits the remyelination capacity of OPCs and oligodendrocytes. OPC RNA sequencing revealed that the mTOR pathway plays a substantial role in remyelination failure. This pathway can be manipulated by caloric restriction or by administration of the AMPK‐agonist metformin to reverse the decline in OPC differentiation and restore their ability to remyelinate axons.^5^ Moreover, multiple OPC differentiation inhibitors, including PSA‐NCAM, Lingo‐1, Jagged, and Galectin‐4, appear relatively overexpressed. Additionally, IFN‐γ, Gli1, and Sirt1 inhibited the proliferation and differentiation of NSCs in demyelinating lesions.^34^ Remyelination failure leads to axonal loss and neurodegenerative changes over time. Therefore, specific targeting of this pathological process is expected to deliver a breakthrough in MS treatment in the future.

## 3 FGF/FGFR SYSTEM

### 3.1 FGFs, FGFRs, and co‐receptors

**FIGURE 1:**
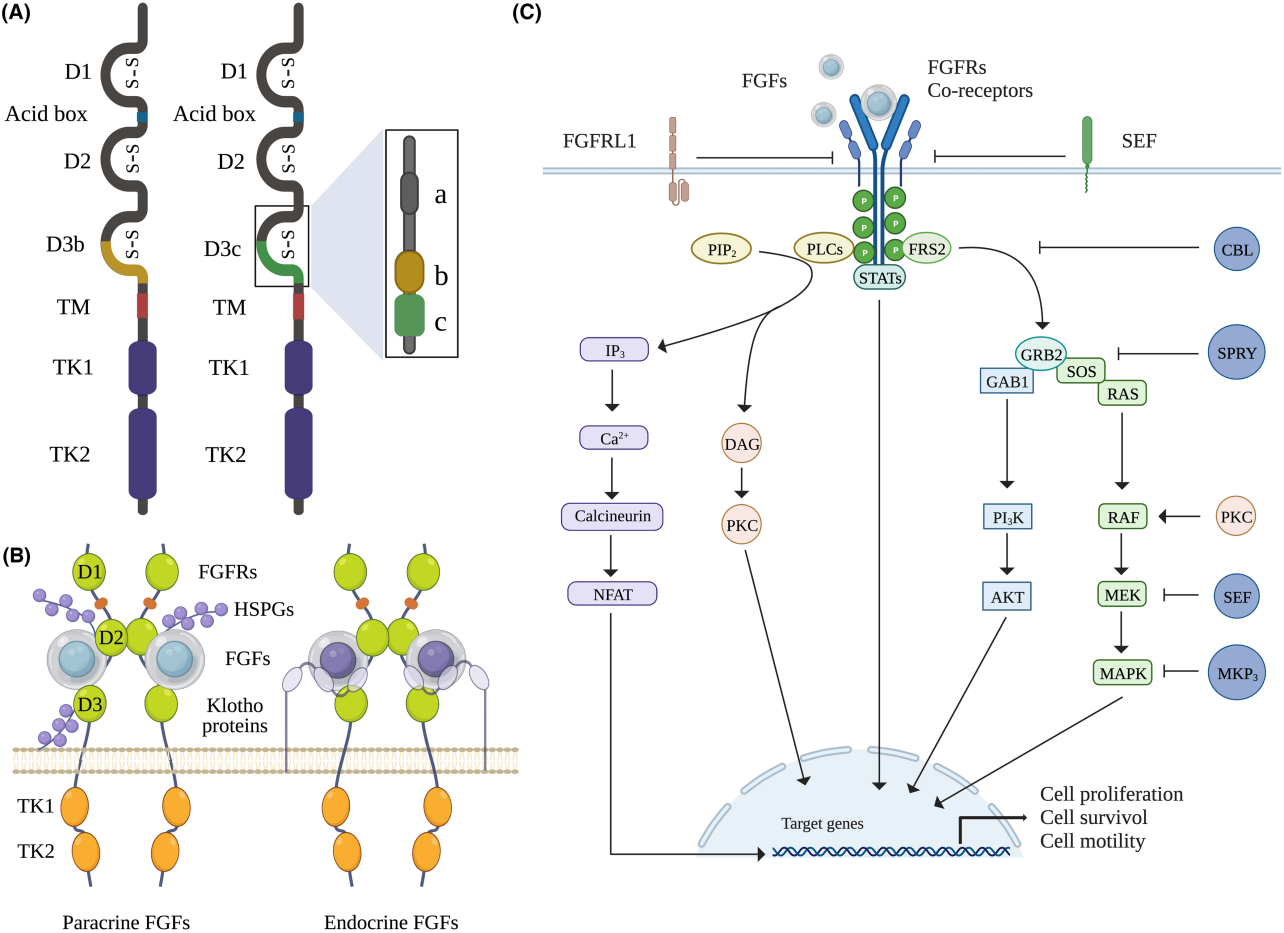
The FGF/FGFR signaling system. (A) FGFR monomer structures: FGFR is a form of the extracellular domains and intracellular catalytic domains linked by a single pass transmembrane domain. Except for FGFR4, the other three FGFR coding genes generate two major splice variants in D3, termed as D3b and D3c, which are essential determinants of ligand binding specificity. (B) The relative orientation of the FGF/FGFR/co‐receptor complex. (C) The downstream pathways of FGF/FGFR signaling: binding of FGFs triggers the dimerization and activation of FGFRs. Activated FGFRs phosphorylate FRS2, which binds to SH2 domain‐containing adaptor GRB2 and GRB2 will subsequently bind to SOS and GAB1 to activate RAS/RAF/MAPKs pathway, including ERK, p38 and JNK, as well as the PI3K/AKT pathway. Independent of the FRS2 binding, FGF signals also activate STATs and PLCγ. Activated PLCγ hydrolyzes PIP2 to DAG and PIP3, which stimulates calcium release from the endoplasmic reticulum and activation of calcium/calmodulin dependent protein kinases. FGFRL1 and SEF are transmembrane proteins and can interact directly with FGFRs to negatively regulate it. Phosphorylation of the MAPK/ERK cascade can be negatively regulated by SEF. SPRY acts at the level of Grb2 to attenuate FGF/FGFR signaling. MKP3 functions as a negative regulator by affecting the phosphorylation of the ERK. AKT, protein kinase B (AKT); DAG, diacylglycerol; ERK, extracellular signal regulated kinase; FRS2, FGFR substrate 2; GAB1, GRB2 associated binding protein 1; JNK, c‐Jun N‐terminal kinase; MAPK, mitogen activated protein kinase; PI3K, phosphatidylinositol 3‐kinase; PKC, protein kinase C; PLCγ, phospholipase Cγ; SOS, son of seven.

The 18 FGFs cluster into six subfamilies, with the FGF1, FGF4, FGF7, FGF8, and FGF9 subfamilies functioning in a paracrine manner and the FGF19 subfamily members operating as endocrine factors.^11^ FGFRs contain a single transmembrane helix (termed TM), three extracellular immunoglobulin‐like domains (termed D1‐3), and two intracellular tyrosine kinase domains (termed TK1‐2). An eight‐residue acid box, a hallmark of FGFRs, is located between D1 and D2 and, together with the D1 loop, plays an autoinhibitory role in receptor activation.^36^ The FGF‐binding region is in D2 and interacts with D3 providing specificity. Two alternative splice sites (D3b and D3c) of the D3 protein show distinct FGF binding specificities (Figure 1A). The FGFR D3b isoform is commonly seen in epithelial cells, whereas the FGFR D3c isoform is typically found in mesenchymal cells. Although FGFR1‐3 exhibits frequent alternative splicing, there is no isoform due to the absence of alternative splicing exons in FGFR4.^36^, ^37^ Depending on the combination with co‐receptors, which include heparin sulfate (HS)/proteoglycans (HSPGs) and Klotho proteins, can FGFs‐FGFRs binding elicit a signal. Most of FGFs feature HS binding domains, and HSPGs are widely distributed in the extracellular matrix. Their different affinities determine whether they work in a paracrine, autocrine, or endocrine way.^11^, ^38^ Not only can HSPGs tether FGFs and enable them to function in an autocrine or paracrine way, but they also enhance FGFS signaling by forming FGF/FGFR/HSPG complexes.^39^ In contrast, the endocrine FGF subclass ligands (FGF19, FGF21, and FGF23), with a weak affinity for HSPGs, utilize Klotho proteins as co‐receptors for binding to their respective FGFRs^37^, ^40^ (Figure 1B). However, they exhibit a strong affinity for FGFR/Klotho complexes but a limited affinity for individual FGFRs or Klotho proteins.^41^ Klotho proteins are a class of transmembrane proteins consisting of the α‐, β‐, and γ‐Klotho subunits. α‐Klotho is necessary for the activity of FGF23, and the biological effects of FGF19 and FGF21 need β‐Klotho (Table 1).

**TABLE 1:** Classification of FGF ligands and their corresponding receptors.

Function manner	FGF subfamily	FGFs	FGFRs	Co‐receptors
Paracrine FGFs	FGF1	FGF1 (aFGF)	All FGFRs	HSPGs
		FGF2 (bFGF)	FGFR1b, FGFR1c, FGFR2c, FGFR3c, FGF4	
	FGF4	FGF4	FGFR1c, FGFR2c, FGFR3c, FGF4	
		FGF5	FGFR1c, FGFR2c	
		FGF6	FGFR1c, FGFR2c, FGFR3c, FGF4	
	FGF7	FGF3	FGFR1b, FGFR2b	
		FGF7 (KGF)	FGFR1b, FGFR2b	
		FGF10	FGFR1b, FGFR2b	
		FGF22	FGFR1b, FGFR2b	
	FGF8	FGF8	FGFR1c, FGF2c, FGFR3b, FGFR3c, FGFR4	
		FGF17	FGFR1c, FGF2c, FGFR3b, FGFR3c, FGFR4	
		FGF18	FGFR1c, FGF2c, FGFR3b, FGFR3c, FGFR4	
	FGF9	FGF9	FGFR1c, FGF2c, FGFR3b, FGFR3c, FGFR4	
		FGF16	FGFR1c, FGF2c, FGFR3b, FGFR3c, FGFR4	
		FGF20	FGFR1c, FGF2c, FGFR3b, FGFR3c, FGFR4	
Endocrine FGFs	FGF19	FGF15/19	FGFR1c, FGF2c, FGFR3c, FGFR4	β‐Klotho
		FGF21	FGFR1c, FGFR3c	
		FGF23	FGFR1c, FGFR3c, FGFR4	α‐Klotho
Intracellular FGFs	FHFs	FGF11		
		FGF12		
		FGF13		
		FGF14		

Abbreviations: FGF, fibroblast growth factor; FGFR, fibroblast growth factor receptor; HSPGs, heparin sulfate (HS)/proteoglycans.

### 3.2 FGF/FGFR signaling

Upon ligand binding, FGFRs dimerize and cause the phosphorylation and activation of intracellular tyrosine kinases.^42^ FGFR substrate 2 (FRS2), a key adaptor protein, is phosphorylated by activated FGFRs, which causes the activation of four intracellular pathways: the phospholipase Cγ (PLCγ) signal; signal transducer and activator of transcription (STAT) signal; phosphatidylinositol 3‐kinase (PI_3_K)/protein kinase B (AKT) signal; and mitogen‐activated protein kinase (MAPK) signal.^43^, ^44^, ^45^ Moreover, FGFR‐like 1 (FGFRL1),^46^ SPRY, CBL, SEF, MAPK phosphatase 3 (MKP_3_), and MKP_1_ can also negatively regulate FGF/FGFR signaling to varying extents. These regulatory factors modulate intracellular signaling or ligand binding^42^ (Figure 1C).

## 4 ROLE OF FGF/FGFR SIGNALING IN CNS

FGF/FGFR signaling not only plays a crucial role in CNS formation during development but also has a broad function in the adult CNS. A prototype member, FGF1, is expressed by neurons in adult neural tissue and acts as a mitogen in neurodevelopmental processes.^47^, ^48^, ^49^ FGF1 stimulated NSC cell expansion and neurite outgrowth in neurons.^50^, ^51^ In vitro and in vivo, FGF2 controls NSC proliferation. Under the induction of FGF2, it has been discovered that undifferentiated precursor cells in the adult mouse proliferated and differentiated into various CNS cells, such as neurons, oligodendrocytes, and astrocytes.^52^ Several studies have shown that FGF2 is also crucial for NSC proliferation in vivo. Granule precursor neuron proliferation was four times higher after subcutaneous injection of FGF2 and had a 250% increase in the subventricular zone of the lateral ventricles. Furthermore, a 68% and 50% increase in DNA synthesis in hippocampal and whole cerebellar homogenates were observed following in vivo FGF2 treatment.^53^ Moreover, the generation and dendritic development of new dentate granule cells was also enhanced after intracerebroventricular FGF2 infusion.^54^ However, the positive regulatory effect of FGF2 on neuronal proliferation was reversed after treatment with FGF2‐specific neutralizing antibodies.^55^ These results indicate that FGF2 is an important neurogenic factor that directly acts on the mitosis of NSCs to promote their proliferation. Conversely, according to multiple studies, FGF2 is not necessary to proliferate neuronal precursors, as FGF2‐deficient mice show normal neural progenitor proliferation during development. However, these mice exhibited partial cerebral cortex loss, impaired neural stem cell differentiation, and increased CNS cell apoptosis, suggesting that FGF2 triggers neural progenitors to migrate and differentiate.^56^, ^57^ In addition, other FGFs, such as FGF4, FGF8, FGF9, FGF10, and FGF21, regulate neuronal fate.^58^, ^59^, ^60^, ^61^, ^62^

The CNS myelin‐producing cells, oligodendrocytes, play a central role in generating and preserving the pace and the power of axonal electrical impulses. Neurological deficits in MS result from myelin damage or insufficient remyelination. Understanding the signals involved in developing oligodendrocyte‐driven myelination may shed light on demyelinating disease prevention and treatment. OPCs migrate to various brain regions during development, transforming into myelin‐producing cells.^63^, ^64^ Several growth factors, including FGFs, control the development of oligodendrocytes.^65^ However, the effect of FGF/FGFR signaling on oligodendrocyte development is regulated by the differential expression of FGFRs. Early and late OPCs express FGFR3, FGFR2 is expressed in mature oligodendrocytes, and both express FGFR1 but not FGFR4.^66^, ^67^ Each FGFR has different roles and focuses during the development and maturation of oligodendrocytes. FGFR1 may transduce signals that stimulate early OPCs proliferation and migration, while FGFR3 signaling primarily controls the late OPCs transformation to oligodendrocytes, and FGFR2 is mainly involved in modulating oligodendrocyte differentiation and myelination.^65^ FGFR1 and FGFR2 in CNS myelination are crucial since they initiate myelination, regulate myelin thickness independently of oligodendrocyte differentiation, and contribute to the remyelination of chronically demyelinated lesions.^68^ Insufficient myelin protein production by oligodendrocytes in FGFR1/FGFR2‐double knockout mice prevents myelination from reaching average levels of myelin thickness.^68^, ^69^

Furthermore, ERK1/2 and PI_3_K/AKT/mTOR, the downstream mediators of these FGFR signaling pathways, sequentially regulate myelination by affecting distinct stages of the oligodendrocyte lineage. ERK1/2 signaling regulates both the transition from early to late OPCs and subsequent immature oligodendrocyte stages. The mTOR signaling pathway is necessary to transform from immature to mature oligodendrocytes.^67^, ^68^ There are many members of the FGF family, each with distinct roles in myelination. FGF2 promotes OPC migration and proliferation but prevents them from differentiating at the terminal stage. FGF2 also affects mature, post‐mitotic oligodendrocytes and causes increased process elongation through FGFR2 stimulation and decreased re‐entry into the cell cycle and myelin proteins via FGFR1.^65^, ^70^ FGF8 and its related subfamily member FGF17 target OPCs and selectively activate FGFR3 to inhibit their differentiation. However, FGF9 stimulates myelination through the specific activation of FGFR2 in differentiated oligodendrocytes.^71^ FGF18 exerts corresponding functions by activating FGFR3 and FGFR2 in OPCs and differentiated oligodendrocytes, respectively.^70^ Circulating FGF21 has been shown to enhance OPC proliferation in both in vivo and in vitro studies, and this was reliant on β‐Klotho presence.^9^, ^62^ However, under normal circumstances, FGF21 transport into the CNS is limited, as the amount of FGF21 in healthy individuals' cerebrospinal fluid (CSF) is approximately 60% lower than that in the peripheral circulation.^72^ Nonetheless, when the BBB is damaged, FGF21 penetrates the CNS to directly promote myelination. Moreover, FGF21 also regulates the expression of the VEGF2 receptor, which modulates OPC migration and indirectly affects oligodendrocyte development and remyelination.^9^

Immunity is continually regulated by FGF signaling, which is, in turn, modified by immune cells during inflammation and tissue healing. FGF signaling cascades are important in CNS inflammatory responses by modulating immune homeostasis and host defense. FGF1 reduces the inflammatory response associated with neuropathic pain by inducing the production of the Th2 cytokine IL‐4, upregulating arginase‐I (Arg‐I), and suppressing the activation of microglia and astrocytes.^73^ In a study conducted by Forouzanfar et al.,^73^ FGF1 treatment reduced the ratios of Bcl2, cleaved caspase 3, MMP‐2, IL‐1β, and Iba1 in model animals of chronic sciatic nerve contractile injury, and modulated apoptosis and neuroinflammation during treatment of neuropathic pain. Both the production of FGF2 protein and the phosphorylation of downstream molecule ERK were restrained in a neuroinflammation‐induced depression model. However, exogenous infusion of FGF2 not only prevented the reduction in ERK1/2 phosphorylation in neuroinflammatory states but also inhibited the expression of proinflammatory cytokines like IL‐1β, IL‐6, and TNFα while increasing the amount of the anti‐inflammatory cytokine IL‐10. These responsive changes reversed depressive‐like behaviors and neuroinflammation‐induced impairment of hippocampal neurogenesis and were blocked by FGFR inhibitors.^74^, ^75^ Notably, FGF2 signaling has demonstrated immune regulatory actions in the aging brain by re‐establishing the balance of proinflammatory cytokines.^74^ Furthermore, FGF2 supplementation attenuated various inflammatory parameters in spontaneous epileptic lesions, with IL‐1β, whose expression was almost entirely blocked, appearing to have the greatest effect.^76^ FGF9, another member of the FGF family, recruits macrophages. Studies have elucidated that FGF9 signaling promoted inflammation and neuronal apoptosis by affecting the stimulation of M1‐type macrophages and the ERK signaling pathway. In contrast, knockdown of the FGF9 gene inhibited macrophage recruitment, thereby attenuating nerve damage.^77^ Controlling inflammatory responses is thought to be a viable therapeutic approach for stroke. Like other FGFs, after a stroke, recombinant human FGF21 has anti‐inflammatory properties that attenuate inflammatory cell polarization and the infiltration of peripheral immune cells, showing its potential as an anti‐inflammatory agent in stroke.^78^ Taken together, FGF/FGFR signaling presumably exerts a pivotal role in neural tissue regeneration, remyelination, and neuroinflammation.

## 5 EXPLORATION OF FGF/FGFR IN MS

### 5.1 FGF1 subfamily

In MS, FGF1 is predominantly expressed in remyelinated lesions, with its production being lower in the demyelinated lesion core than in the remyelinated rim.^79^ In cerebellar slice cultures, FGF1 promoted remyelination and directly accelerated myelination. Furthermore, by inducing the upregulation of the leukemia inhibitory factor (LIF) and the chemokine CXCL8 in human astrocytes, which are involved in the recruitment of oligodendrocytes, FGF1 indirectly promoted the induction of remyelination.^79^ LIF has been demonstrated to support oligodendrocyte development and survival,^80^, ^81^, ^82^ in addition to promoting myelination.^83^ In EAE, it has also been proven to prevent oligodendrocyte death^84^ and promote remyelination.^85^ Astrocytes at the edges of active MS lesions secrete high levels of CXCL8, thereby recruiting OPCs into the lesions and participating in their regeneration.^86^, ^87^

How FGF2 affects oligodendrocyte responses during demyelination and remyelination in MS is debatable. On one hand, FGF2 is regarded as a neuroprotective agent that promotes remyelination in MS.^17^ FGF2 has mainly been detected in microglial and macrophages of active, chronic‐active, and chronic‐inactive lesions in MS.^88^ An increase in serum FGF2 levels was also found in gadolinium‐enhanced lesions in RRMS and disability progression of SPMS.^89^ However, FGF2 peaked in the initial stage of remyelination.^90^ Consistently, ciliary neurotrophic factor (CNTF) expression was induced around remyelination lesions of MHV‐A59‐infected model mice, and FGF2 and its receptor were induced in spinal cord astrocytes after CNTF injection, suggesting that CNTF acts through the FGF2/FGFR pathway.^91^ Endogenous FGF2 activity was tested in glial cultures purified from demyelinated lesions of MHV‐A59‐infected mice, and these experiments demonstrated its ability to act as an effective mitogen related to the OPC proliferative response in demyelinating and remyelinating tissues.^92^ FGF2 null EAE presented intensive clinical symptoms compared to normal EAE,^17^ and FGF2 gene therapy reverted this phenomenon^93^ and significantly reduced the infiltration of myelotoxic cells into the CNS. Moreover, mice overexpressing the FGF2 gene showed increased OPCs and myelin‐forming numbers in demyelinating regions.^93^ Indeed, FGF2 null mice had noticeably more degenerative nerve fibers and axonal loss. Concurrently, there was a considerable reduction in the quantity of remyelinated axons.^17^ On the other hand, it has been suggested that FGF2 is a negative factor in both the myelination processes and remyelination failure. In the cuprizone‐induced mouse model, FGF2 expression levels were increased,^94^ and high FGF2 levels induced marked destruction of mature oligodendrocytes and severe myelin loss in the CNS.^95^ FGF2 knockout promoted oligodendrocyte regeneration by spontaneously enhancing the proliferation of OPCs in vivo during the lesion recovery phase, thereby promoting remyelination^94^ and reducing axonal atrophy in demyelinating lesions.^96^ Stimulation of lesion‐derived glial cells in MHV‐A59‐infected mice showed that high concentrations of FGF2 promoted OPC proliferation. In contrast, OPC development into oligodendrocytes was favored in vitro by attenuating endogenous FGF2 via neutralizing antibody.^97^ Indeed, these outcomes align with the improved OPC differentiation observed in FGF2^−/−^ animals. The role of FGF2 is variable since chronically high FGF2 levels in the CSF reverse the positive benefits, and the secretion of FGF2 by immune cells increases with disease progression in MS/EAE.

### 5.2 FGF8 subfamily

FGF8, FGF17, and FGF18 belong to the FGF8 subfamily. They share 60 to 80 percent of amino acid sequence similarity and have comparable receptor binding features.^98^ FGF8 is an original factor that, consistent with FGF2 roles, induces OPC activation, migration, and proliferation but does not hamper differentiation. In the FGF8‐treated medium, a remarkable increase in the number of OPCs was detected, and they displayed both immature and mature oligodendrocyte markers. Therefore, it appears that FGF8 stimulates OPC proliferation without inhibiting differentiation, ultimately producing more mature oligodendrocytes. It was observed that FGF8 bound to FGFRs could attract OPCs and induce their migration, which was also recapitulated in animal models for demyelination.^99^ When a demyelinating lesion arises, OPCs that are present throughout life in the CNS are prepared to differentiate into mature oligodendrocytes.^100^ However, dysfunction of OPC activation in MS affects remyelination processes.^101^ A reduced capacity for myelination may be caused in part by the attenuation of OPC migration and differentiation, positioning FGF8 as a potential therapeutic target.

### 5.3 FGF9 subfamily

The cascade of pathogenic events in MS eventually leads to the loss of neurons and axons, which can be measured by reduced brain volume on volumetric magnetic resonance imaging (MRI) in vivo. Between 0.5% and 1.5% of MS patients develop brain atrophy each year, and during the progressive stages of the disease, the deep gray matter structures display a higher rate of degeneration.^2^ It was shown that plasma FGF9 levels are strongly associated with brain volume loss in MS patients, and the annual percentage of brain volume change was inversely related to these levels.^102^ FGF9 was high in early active lesions and was upregulated in ongoing lesions of MS patients with longstanding progressive disease. However, FGF9 levels were remarkably lower in healthy white matter and almost nonexistent in chronically demyelinated inactive lesions.^18^ Furthermore, GFAP+ astrocytes and OLIG2+ and NOGO‐A+ oligodendrocytes were shown to produce FGF9. This suggests that FGF9 was induced by a localized glial response toward ongoing tissue damage in MS. Although FGF9 was discovered to prevent OPCs from developing into mature oligodendrocytes, the authors did not agree that this direct effect was responsible for the inhibition of remyelination. Rather, FGF9 still serves as an OPC stimulant and contributes to the generation of proteolipid protein+ (PLP+) oligodendrocytes in the complex cellular environment.^18^ However, this proliferation is of little importance since FGF9 inhibited the differentiation of precursor oligodendrocytes into mature myelination‐competent oligodendrocytes through an astrocyte‐dependent mechanism. Furthermore, FGF9 also enhanced the expression of chemokines CCL2 and CCL7,^18^ which are known to be expressed in MS lesions and recruit macrophages and microglia to initiate the inflammation of MS. The dual pathogenic role of FGF9 in MS, which involves both triggering a proinflammatory response and inhibiting remyelination, has significant implications for the etiology of the disease.

### 5.4 FGF19 subfamily

The endocrine FGF subclass ligands (FGF19, FGF21, and FGF23) are also known as the FGF19 subfamily. FGF21, mainly released by the skeletal muscle, pancreas, liver, kidney, and adipose tissue, exerts pleiotropic effects in regulating glucose, lipid, and energy homeostasis.^103^ Apart from metabolic regulation, it has been recently discovered that FGF21 is secreted by neurons and exhibits neuroprotective functions.^104^ FGF21 was dramatically downregulated after cerebral ischemia, and the upregulation could restore brain function by reducing cerebral infarction and ameliorating neuronal cell death.^105^ Moreover, FGF21 promoted remyelination after traumatic brain injury.^103^ In the lysophosphatidylcholine‐induced demyelination model, treatment with a neutralizing antibody against FGF21 or gene knockout abolished these positive effects on OPCs.^9^ The effect of FGF21 on OPCs seems to be limited to promoting proliferation without affecting their differentiation or apoptosis. Furthermore, FGF21 did not modulate the cell fate of astrocytes, and Kuroda et al. detected no significant proliferation of OPCs cultured in astrocyte supernatant with FGF21 pre‐treatment, indicating that FGF21 acts directly as an OPC mitogen. Interestingly, FGF21‐mediated proliferation of human OPCs in autopsy samples from MS patients has been observed.^9^ Therefore, we can infer that FGF21‐mediated OPC proliferation and consequent remyelination are conserved in CNS demyelinating models.

FGF23 was first identified in the ventrolateral thalamic nucleus of the mouse brain, and its physiological role has recently attracted significant attention.^106^ The FGF23 protein is present in three distinct forms in the bloodstream: a full‐length mature form and two inactive (C‐ and N‐terminal) fragments.^107^ It is generally accepted that intact FGF23 is a bioactive factor that controls the metabolism of phosphate and vitamin D. In contrast, high levels of the inactive FGF23 forms have been demonstrated to inhibit these effects.^108^ Considered a bone‐derived hormone, FGF23 is primarily released by osteocytes and osteoblasts in the skeleton. It is a component of the novel hormonal bone–parathyroid–kidney axis, which interacts to form hormonal homeostasis.^109^, ^110^ Several studies have shown that serum FGF23 concentrations were elevated in RRMS patients. However, calcitriol levels were reduced, indicating that elevated FGF23 levels in MS may disrupt the FGF23‐PTH‐vitamin D axis, resulting in pathological effects.^19^, ^111^ However, in a study that measured plasma levels of FGF23 in 91 MS patients and 92 healthy controls, no difference was observed.^112^ Similar results were obtained in a study by Alagha et al.^113^ In addition, they also found that the secretion of FGF23 in the CSF of MS patients was comparable to that of the healthy population. There are several reasons for these diverging results: (1) The study of elevated FGF23 was conducted in RRMS, and the latter two studies were the result of a mixture of all clinical subtypes of MS. Furthermore, the pathogenesis of MS subtypes differs, which can lead to different outcomes; (2) As mentioned earlier, FGF23 exists in two forms, an intact active form, and an incomplete form. Of the studies that concluded that FGF23 was elevated, intact FGF23 was measure, while the other studies measured all forms of FGF23, which may have biased the results; (3) The experimental samples and methods used in these studies differed slightly, which may also cause inconsistent results. The elevated FGF23 in MS patients inhibited 1‐α‐hydroxylase and upregulated 24‐α‐hydroxylase to eliminate 1,25‐(OH)_2_D_3_ levels.^111^ Previous studies have demonstrated that vitamin D has an immunomodulatory effect and promotes the multiplication of NSCs and their differentiation into mature neurons and oligodendrocytes.^114^ FGF23 might be involved in immune responses via suppression of vitamin D production and direct interaction with immune cells such as macrophages and dendritic cells (DCs). Under the stimulation of LPS/IFN‐γ, activated DCs and macrophages contribute to the increased serum FGF23 levels, mediated by the nuclear factor‐κB and the JAK/STAT1 signaling pathways.^115^ Compelling studies suggest that FGF23 targets macrophages, which express FGFR1 along with DCs, and exhibit increased α‐Klotho expression upon inflammatory stimulation.^116^ The interaction of FGF23 with the FGFR1/α‐Klotho complex affects the polarization of macrophages to the M1 phenotype, blocks the transition to the M2 phenotype and induces M1‐type macrophages to secrete TNFα and suppress Arg‐I expression in M2 macrophages, resulting in sustained inflammation.^115^, ^116^ However, 1,25‐(OH)_2_D_3_ has been reported to have the opposite effect on cytokine secretion of macrophages.^116^ FGF23 has a proinflammatory function and counters the regulatory action of 1,25‐(OH)_2_D_3_ on immune responses. Accordingly, it is conceivable that elevated FGF23 levels contribute to MS by promoting inflammation.

### 5.5 Fibroblast growth factor receptors

**FIGURE 2:**
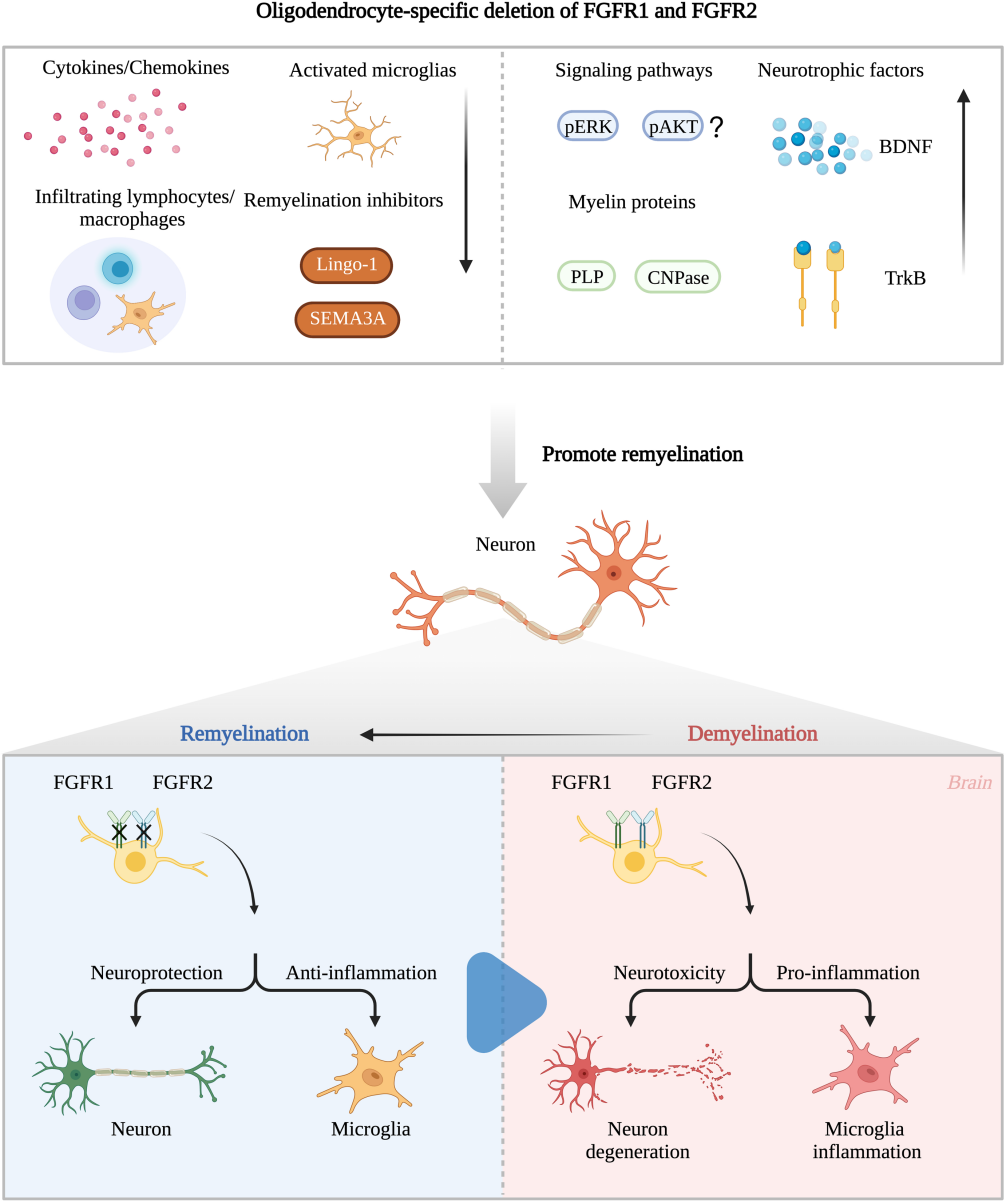
Role of oligodendrocyte‐specific deletion of FGFR1 and FGFR2. Pro‐remyelination and anti‐inflammatory mechanisms of oligodendrocyte‐specific deletion of FGFR1 and FGFR2 in EAE. BDNF, brain derived neurotrophic factor; CNPase, 2′,3′‐cyclic‐nucleotide 3′‐phosphodiesterase; EAE, experimental autoimmune encephalomyelitis; PLP, Proteolipid protein; SEMA3A, Semaphorin 3A.

To characterize the role of FGFRs in oligodendrocytes in vitro, researchers used the multi‐kinase inhibitor and FGFR1‐3 inhibitor to block FGFR signaling. The application of inhibitors increased the expression of the myelin‐associated proteins PLP and 2′,3′‐cyclic‐nucleotide 3′‐phosphodiesterase. Furthermore, upregulation of neurotrophic factor BDNF/TrkB signaling and decreased expression of the remyelination inhibitor semaphorin 3A were observed. These effects may be related to the reduction in downstream molecules of the FGF/FGFR signaling pathway, such as ERK and AKT phosphorylation, in oligodendrocytes.^117^ These findings have been confirmed in vivo. Conditional ablation of FGFR1 or FGFR2 in oligodendrocytes alleviated the symptoms of motor deficits in MOG35‐55‐induced EAE.^14^, ^16^, ^118^ In the spinal cord of FGFR1^ind−/−^ and FGFR2^ind−/−^ animals, myelin, axonal loss, and the infiltration of inflammatory cells were decreased in the chronic phase of EAE without causing any alterations in the acute phase. The protective effects of the oligodendrocyte‐specific deletion of FGFR1 and FGFR2 on EAE are mainly manifested in two aspects. First, the knockdown of FGFRs can regulate the inflammatory environment in the CNS. T cells and B cells, as well as macrophages and activated microglia, were significantly reduced in the spinal cord and cerebellum of FGFR1^ind−/−^ mice, along with the decreased expression of the proinflammatory cytokines TNFα, IL‐1β, and IL‐6, and the chemokine CX3Cl1 and its receptor CXC3R1.^14^, ^118^ Furthermore, FGFR2^ind−/−^ mice showed a similar phenotype,^16^ and this low‐inflammatory environment facilitates remyelination and tissue repair of EAE lesions. Second, the deletion of FGFR1 and FGFR2 exhibited robust pro‐remyelination activity. Although the depletion of FGFR1 and FGFR2 did not affect the numbers of cells from the oligodendrocyte lineage, the high expression of pERK, pAKT, BDNF, and TrkB, and the low level of Lingo‐1 in the spinal cord was caused by specific knockdown of FGFR1.^14^, ^118^ The phenotype of FGFR2^ind−/−^ mice varied slightly, with an increase in PLP‐positive cells in the spinal cord and a decrease in the expression of SEMA3A. At the same time, there were no significant increases in pERK or TrkB,^16^ indicating that the two FGFR receptors in the EAE model mediate different effects (Figure 2). In the cuprizone demyelination model, acute‐phase demyelination and myelin recovery were not affected by oligodendrocyte‐specific deletions of FGFR1 and FGFR2. However, in the chronic period, double knockout of FGFR1 and FGFR2 caused remyelination failure.^119^ Additionally, single deletion of FGFR1 promoted remyelination and functional recovery in the chronic phase.^15^ Therefore, these data suggest that FGFR signaling inhibits regenerative processes, particularly chronic demyelination.

## 6 SUMMARY

Current MS treatments have achieved near complete control over RRMS and focal neuroinflammation. However, no effective treatment strategies are available concerning neurodegeneration and disability. FGF signal transduction is involved in the development of the CNS, enhances the proliferation and axonal growth of NSCs in adults, and promotes their differentiation into various CNS cells, such as neurons and oligodendrocytes. Furthermore, the development of oligodendrocytes is inseparably linked to FGFs, and distinct FGFRs are expressed at different stages of their development; therefore, FGFs play pivotal roles at each stage. Surprisingly, several studies have shown that neuroinflammation is regulated partly by FGF signaling, as FGF1, FGF2, and FGF21 have anti‐inflammatory functions, while FGF9 induces neuroinflammation. These results all emphasize the importance of FGF/FGFR signaling in the pathogenesis of MS. Some FGFs are mainly expressed in acute demyelinating lesions and mediate disease progression. Others display high concentrations in remyelinating lesions in the chronic phase of MS and promote the life cycle of OPCs and the maturation of oligodendrocytes, which helps to enhance tissue repair and restore motor function (Table 2). However, due to the large size of the FGF family and their far‐spanning actions, coupled with their ambiguous roles in MS and their evolution through disease progression, it is difficult to explore their current therapeutic utility for MS. Furthermore, before FGFs become relevant biomarkers for monitoring MS disease course and disease identification, various conditions need to be met: the role of other members of the FGF family in MS and the correlation between FGFs and clinical data, including MS subtypes, clinical features, and disease stages, require further investigation.^120^ In addition, more research is needed to detect the sensitivity, specificity, and stability of FGFs as biomarkers and they must be compared with existing oligoclonal bands and IgG to clarify their characteristics.^121^

**TABLE 2:** Variety actions of FGFs in MS.

FGFs	Concentration in MS	Roles in remyelination	Roles in neuroinflammation
FGF1	Predominantly expressed in remyelinated lesions; lower in the demyelinated lesion core compared to the remyelinated rim	Upregulates expression of LIF and CXCL8 in astrocytes to support oligodendrocyte maturation, differentiation, and survival	Reduces the inflammatory response via inducing the expression of IL‐4, Arg‐I and inhibiting the activation of microglia and astrocytes
FGF2	Mainly detected in active lesions and ambitus of chronic‐active as well as chronic‐inactive lesions	Enhances the proliferation and survival of OPCs in the early stage, and inhibits their differentiation into oligodendrocytes after prolonged stimulation	Prevents the expression of proinflammatory cytokines such as IL‐1β, IL‐6, and TNFα and increases the anti‐inflammatory cytokine IL‐10 levels
FGF8	Not detected	Induces OPCs' activation and migration	Not yet identified
FGF9	Highly expressed in early active lesions, and upregulated in ongoing lesions; strongly associated to loss of brain volume in MS patients	Inhibits the differentiation of isolated OPC into mature oligodendrocytes	Enhances the expression of proinflammatory genes, such as chemokines CCL2 and CCL7
FGF21	Not detected	Mediates OPCs proliferation and leads remyelination	Attenuates inflammatory cell polarization and infiltration of peripheral immune cells into CNS
FGF23	Serum concentration elevates; no significant increasement in CSF	Not yet identified	Counters the regulatory action of 1,25‐(OH)_2_D_3_ on immune responses; affects the polarization of macrophages to the M1 phenotype and induces the secretion of TNFα, blocks the transition to M2 macrophages and the expression of Arg‐I

Abbreviations: CSF, cerebrospinal fluid; FGF, fibroblast growth factor; LIF, leukemia inhibitory factor; MS, Multiple sclerosis; OPC, oligodendrocyte precursor cell.

In contrast, FGFRs have shown promising therapeutic potential. FGFRs are widely expressed in CNS and immune cells (Table 3) and may be involved in many aspects of MS pathogenesis; therefore, therapies targeting FGFRs can exert multiple effects. Furthermore, conditional deletion of FGFR1 and FGFR2 has improved EAE symptoms, especially in the chronic phase, leading to amelioration of the inflammatory microenvironment, less demyelination, and greater axonal density. The underlying mechanisms may be changes in the levels of ERK and AKT phosphorylation, and the expression of BDNF and several remyelination inhibitors. The increase in BDNF expression is particularly interesting since glatiramer acetate and fingolimod, both currently available MS treatments, have been reported to upregulate BDNF expression; this upregulation may be associated with their therapeutic efficacy.^122^ Based on the findings described here, targeting FGFRs is a promising strategy for treating MS patients.

**TABLE 3:** Cell expression of FGFRs.

FGFRs	Cell type		References
FGFR1	Macrophage T lymphocyte B lymphocyte NK cell	Neuron Microglia Astrocyte OPC Oligodendrocyte	118, 123, 124, 125, 126, 127, 128, 129, 130, 131, 132
FGFR2	Macrophage NK cell	Neuron Microglia Astrocyte OPC Oligodendrocyte	123, 129, 132, 133, 134
FGFR3	Macrophage B lymphocyte	Neuron Microglia Astrocyte OPC Oligodendrocyte	90, 123, 132, 135, 136, 137
FGFR4	Macrophage	Neuron Microglia Astrocyte OPC	123, 132, 138

Abbreviations: FGFR, fibroblast growth factor receptor; OPC, oligodendrocyte precursor cell.

## AUTHOR CONTRIBUTIONS

Zhiguo Chen and Tao Jin designed the study. Qingxiang Zhang wrote the main text, produced the tables and figures. Kaili Zhang was responsible for literature review and data collection. Jie Zhu and Tao Jin critically revised the whole manuscript. All authors read and approved the final manuscript.

## FUNDING INFORMATION

This work was supported by grants from the General Program of the National Natural Science Foundation of China (No. 82171337).

## CONFLICT OF INTEREST STATEMENT

The authors declare that they have no competing interests.

## Data Availability

Data sharing is not applicable to this article as no new data were created or analyzed in this study.
